# Differences in the Pathways of Proteins Unfolding Induced by Urea and Guanidine Hydrochloride: Molten Globule State and Aggregates

**DOI:** 10.1371/journal.pone.0015035

**Published:** 2010-11-30

**Authors:** Olga I. Povarova, Irina M. Kuznetsova, Konstantin K. Turoverov

**Affiliations:** Laboratory of Structural Dynamics, Stability and Folding of Proteins, Institute of Cytology of the Russian Academy of Science, St. Petersburg, Russia; University of South Florida College of Medicine, United States of America

## Abstract

It was shown that at low concentrations guanidine hydrochloride (GdnHCl) can cause aggregation of proteins in partially folded state and that fluorescent dye 1-anilinonaphthalene-8-sulfonic acid (ANS) binds with these aggregates rather than with hydrophobic clusters on the surface of protein in molten globule state. That is why the increase in ANS fluorescence intensity is often recorded in the pathway of protein denaturation by GdnHCl, but not by urea. So what was previously believed to be the molten globule state in the pathway of protein denaturation by GdnHCl, in reality, for some proteins represents the aggregates of partially folded molecules.

## Introduction

Protein unfolding induced by chemical denaturants such as urea and guanidine hydrochloride (GdnHCl) is a common approach to study protein folding in vitro [1]. Meanwhile, it has been shown that low concentrations of GdnHCl can cause protein stabilization by eliminating the strains in protein caused by the electrostatic interactions of charged groups on its surface [2], [3]. Our results also prove it. At low concentration of GdnHCl (around 0.1 M) we have recorded small changes in fluorescence spectrum position and tryptophan fluorescence anisotropy for creatine kinase [4], carbonic anhydrase II (CA II) [5] and increase in chromophore fluorescence intensity of EGFP, DsRed1 and their mutant forms (0.5 M of GdnHCl) [6].

In this work, we have shown that along with the stabilizing effect on protein structure, small amounts of GdnHCl can also cause protein aggregation. The account of this effect clarifies why the transition of some proteins (e.g. α-lactalbumine and CA II) into intermediate state like molten globule is accompanied by blue shift of fluorescence spectrum, increase in anisotropy of intrinsic fluorescence, and increase in fluorescence of hydrophobic dye 1-anilinonaphthalene-8-sulfonic acid (ANS) when denaturation is caused by GdnHCl but not by urea [5], [7], [8]. The increase in ANS fluorescence in the narrow range of GdnHCl concentration for urease is also connected with protein aggregation [9], [10]. For the first time, protein aggregation in the solution of low concentration of GdnHCl we have revealed for actin. In this case the effect is especially pronounced because in aggregation are involved large supramolecular complexes of inactivated actin [11], [12] and it is accompanied not only by the increase in ANS fluorescence intensity, but by the increase in light scattering also.

## Results and Discussion

### Aggregation and changes of inactivated actin surface characteristics induced by GdnHCl

**Figure 1:**
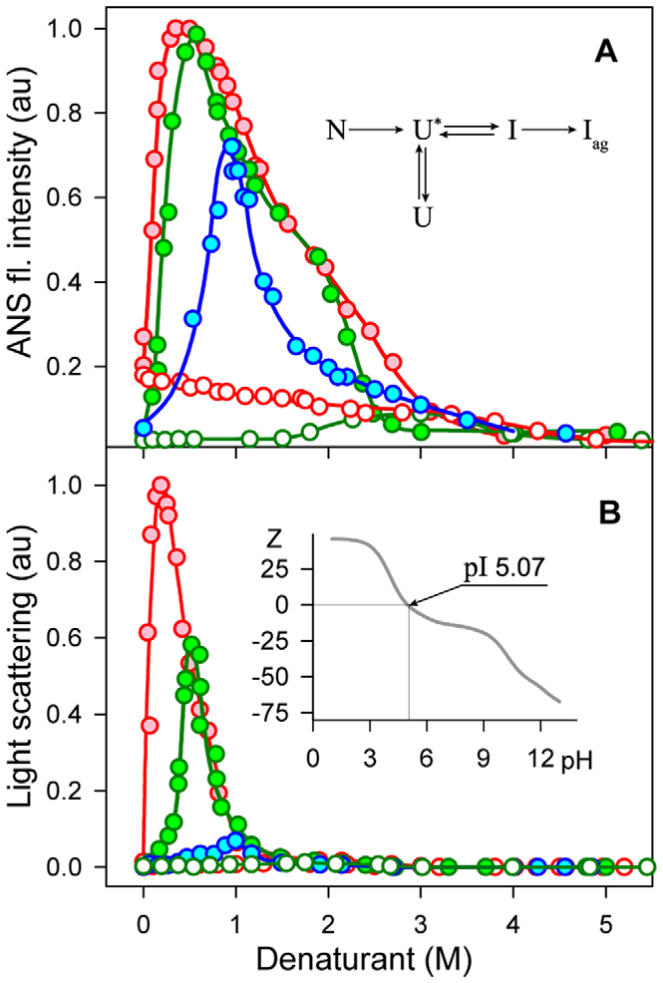
Actin aggregation induced by GdnHCl in low concentrations. The dependence of ANS fluorescence intensity (**A**) and of light scattering (**B**) of the solutions of inactivated actin (red) and initially native actin after 10 min and 24 h of incubation in a solution of denaturants at the appropriate concentration (blue and green) on GdnHCl (closed symbols) and urea (open symbols) concentration. **Insert** in panel **A**. Scheme of actin denaturation and aggregation (N, U^*^ and I are native, essential unfolded and inactivated actin [11], I_ag_ is aggregates of inactivated actin, the details are given in the text). **Insert** in panel **B**. The dependence of the total macromolecule charge of actin on the pH of a solution calculated on the basis of protein amino acid content [24]. The protein concentration was 0.15 mg/ml, ANS concentration was 5⋅10^−5^ M.

The study of actin unfolding – refolding showed that actin denaturation results in the formation of the so-called inactivated actin (I), which represents an ordered aggregate (supramolecular monodisperse complex of 14–16 monomers of partially unfolded actin molecules) with hydrophobic clusters on the surface [11], [12]. The fluorescence intensity of ANS (5⋅10^−5^ M) in the presence of inactivated actin (0.15 mg/ml) is around 20 times greater than in the presence of native actin at the same concentration. This can be explained not only by the existence of hydrophobic clusters on the surface of inactivated actin, but also by the appearance of “hydrophobic pockets” between actin molecules in partially folded state forming inactivated actin. Inactivated actin characteristics are independent of the way of its formation [11], [13]. We decided to examine the characteristics of inactivated actin in solutions of different concentrations of urea and GdnHCl. As it can be expected, the intensity of ANS fluorescence weakly depends on urea concentration up to the concentration when supramolecular complexes are destroyed. However, the dependence of ANS fluorescence on the concentration of GdnHCl was found to be the curve with maximum in the narrow range of small concentrations of denaturant (Figure 1). Furthermore, in the same range of GdnHCl concentrations maximum of light scattering (or even precipitation at high protein concentration) was observed. This means that in this narrow range of GdnHCl concentrations inactivated actin forms large aggregates, and that ANS molecules affinity to these aggregates is very high. ANS incorporates into the hydrophobic pockets between the molecules forming aggregates that result in the dramatic increase in its fluorescence intensity.

We explain protein aggregation by the interactions between the GdnHCl cations (GuH+) and the side chain C = O group of the glutamic acids and glutamine, aspartic acid and asparagine amino acid residues of the molecule. The possibility of such interactions has been shown earlier [14], [15]. In actin, the number of negatively-charged groups from glutamic and aspartic acids (OD2 - 22 groups and OE2 - 28 groups) is greater than that of the positively-charged groups from lysine (NZ – 18 groups), arginine (NH1 – 18 groups) and histidine (NE2 – 9 groups). Therefore, the actin molecule is negatively charged (pI 5.07, see Figure 1A, Insert) at a neutral pH. With an increase in the number of GuH+ ions bound to inactivated actin, the number of positively-charged groups increases, and at some concentration of GdnHCl (0.2–0.3 M), the initially negatively-charged molecules become neutral, which leads to their aggregation. Upon the further increase in GdnHCl concentration, the number of positively-charged groups on the surface of the protein molecules will exceed the number of negatively-charged groups. Therefore, the conditions will no longer be favorable for aggregation. This is the reason for the abrupt decrease in light scattering intensity. The less abrupt decrease in the intensity of ANS fluorescence in comparison with light scattering with the increase in GdnHCl concentration can be explained by the higher affinity of negatively-charged ANS molecules with inactivated actin when it is positively charged, though aggregates are already destroyed.

We have ascertained that native proteins with a pI values at acidic pH, and native actin in particular, do not aggregate at low concentrations of GdnHCl. Due to complex process of actin denaturation and the dependence of the transitions rates upon GdnHCl concentration [11], [12] (see also, Figure 1A, Insert) maximum of ANS fluorescence intensity shifts to lower concentration of GdnHCl with the increase in incubation time. Thus after 24 h of incubation maximum of light scattering and intensity of ANS fluorescence intensity were recorded practically at the same concentrations of GdnHCl as for inactivated actin.

Hydrophobic interactions apparently play a significant role in both inactivated actin formation and in the formation of inactivated actin aggregates in the presence of low concentrations of GdnHCl. As mentioned above, due to existence of hydrophobic pockets in inactivated actin ANS fluorescence intensity in the presence of inactivated actin is 20 times greater than in the presence of native actin. Inactivated actin already has hydrophobic clusters on its surface, but molecules of inactivated actin do not “stick together” because of the negative charges on their surfaces, which prevent this process. At low concentrations of GdnHCl the aggregation of inactivated actin leads to the significant increase in the number of hydrophobic pockets and consequently to the increase in the number of bound ANS molecules, that is recorded by increase in ANS fluorescence. Protein aggregation in the solution of low concentration of GdnHCl is especially pronounced for actin, because in this case large supramolecular complexes of inactivated actin [11], [12] are involved in aggregation.

### Intermediate states in the pathway of CA II unfolding induced by urea and GdnHCl: molten globule and aggregates of molten globules

**Figure 2:**
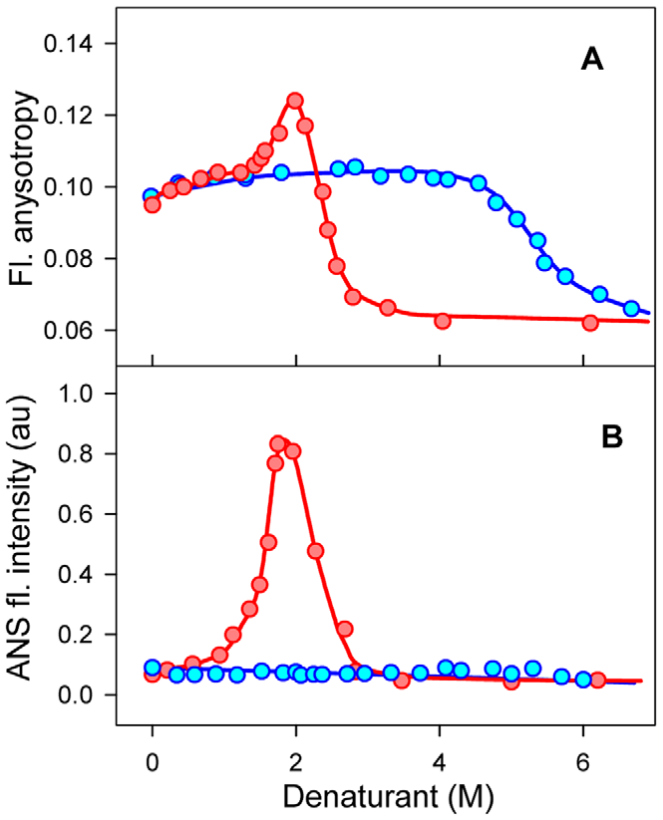
Denaturation of CA II induced by GdnHCl (red symbols) and urea (blue symbols). Panels **A** and **B** represent the changes in anisotropy of intrinsic fluorescence and ANS fluorescence intensity, respectively. The protein concentration was 0.15 mg/ml, ANS concentration was 5⋅10^−5^ M, pH 7.5.

Taking into account the aggregating effect of GdnHCl it became clear why the transition of some proteins into intermediate state like molten globule is accompanied by blue shift of fluorescence spectrum, increase in anisotropy of intrinsic fluorescence, and increase in fluorescence of ANS when protein denaturation is caused by GdnHCl but not by urea [5], [7], [8], [9], [10]. This exactly was observed for CA II [5]. Now it is clear that CA II unfolding is two stage processes both induced by GdnHCl and urea. The appearance of intermediate state of CA II in the pathway of unfolding induced by urea agrees with the results of several works which are reviewed in [16]. The increase of ANS fluorescence intensity is recorded only in GdnHCl solution (Figure 2), because the dye interacts with aggregates of CA II intermediate state. Aggregates of CA II intermediate state are comparative small therefore their formation is seen by the increase of intrinsic fluorescence polarization (Figure 2), but not light scattering.

In conclusion, this work proposes new view on three important points of protein folding: (i) strong chemical denaturant GdnHCl in narrow range of small concentrations, in contrast to urea, can cause aggregation of some proteins in molten globule state; (ii) hydrophobic dye ANS binds with the aggregates of proteins in the molten globule state rather than with the hydrophobic clusters on the surface of a protein in the molten globule state, as was commonly accepted [1], [17]; (iii) for some proteins, what was previously believed to be the molten globule state in the pathway of protein denaturation by GdnHCl in reality represents the aggregates of protein molecules in this state.

## Materials and Methods

Rabbit skeletal muscle actin was purified by one or two cycles of polymerization-depolymerization [18]. The native state of actin was checked by its fluorescence spectrum position characterized by parameter *A* =  (*I*_320_/*I*_365_)_297_, where *I*_320_ and *I*_365_ are fluorescence intensities at λ_em_ = 320 and 365 nm, respectively (λ_ex_ = 297 nm). Actin samples had a parameter *A* value ≥2.53, which corresponds to an inactivated actin content of <4% [19]. The molar extinction coefficient for actin was taken as *E*_280_ = 1.09 (mg/ml)^−1^ cm^−1^ [20]. The final actin concentration varied from 0.1 to 0.44 mg/ml. CA II from bovine erythrocyte was purchased from Sigma (USA) and used without further purification. The extinction coefficient for CA II was taken as *E*_280_ = 1.87 (mg/ml)^−1^ cm^−1^ [7]. The protein concentration varied between 0.05 and 0.5 mg/ml. GdnHCl (Nacalai Tesque, Japan), urea, and ANS (Sigma, USA) were used without additional purification. The concentration of GdnHCl and urea were determined by the refractive index [21] with an Abbe refractometer (LOMO, Russia). The extinction coefficient of ANS was taken as *ε*_350_ = 5000 M^−1^ cm^−1^ [22]. The concentrations of proteins and ANS were determined using a spectrophotometer U-3900H (Hitachi, Japan).

Fluorescence experiments were carried out using a Cary Eclipse spectrofluorimeter (Varian, Australia) and a homemade spectrofluorimeter for registration of fluorescence polarization [23]. Protein intrinsic fluorescence was excited at the long-wavelength edge of absorption spectrum (λ_ex_ = 297 nm) where the contribution of tyrosine residues to the bulk protein fluorescence is negligible. The position and form of the fluorescence spectra were characterized by parameter *A* [19], [23]. The values of parameter *A* and of the fluorescence spectra were corrected by the instrument spectral sensitivity. The fluorescence intensity of the hydrophobic dye ANS was detected at 480 nm (λ_ex_ = 365 nm). The anisotropy of tryptophan fluorescence was calculated by the equation 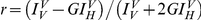, where 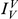 and 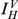 are the vertical and horizontal components of the fluorescence intensity excited by vertically polarized light and G is the relation of vertical and horizontal components of fluorescence intensity excited by horizontally polarized light (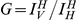) [23]. The intensity of light scattering was detected by the spectrofluorimeter when *λ_reg_* = *λ_ex_*. In the majority of experiments the light scattering was recorded at 297 or 350 nm. The choice of the wavelength did not influence the result.
